# Supplementary material for: Structural Rearrangements of a Dodecameric Ketol-Acid Reductoisomerase Isolated from a Marine Thermophilic Methanogen
Source: Biomolecules. 2021 Nov 11;11(11):1679. doi: 10.3390/biom11111679 (PMC8615647; doi:10.3390/biom11111679)
Supplement: Supplementary file 1 [file biomolecules-11-01679-s001.zip › biomolecules-1442601-supplementary.pdf]

Supplementary Information

Structural rearrangements of a dodecameric Ketol-Acid Reductoisomerase isolated from a marine thermophilic methanogen

Olivier Nicolas Lemaire[1], Marie-Caroline Müller[1], Jörg Kahnt[2] and Tristan Wagner[1*]

Author affiliations:

[1] Microbial Metabolism research group, Max Planck Institute for Marine Microbiology, Celsiusstraße 1, 28359 Bremen, Germany

[2] Core Facility for Mass spectrometry & Proteomics, Max Planck Institute for Terrestrial Microbiology, Karl-von-Frisch-Straße 10, 35043 Marburg, Germany.

* Correspondence: twagner@mpi-bremen.de

Supplementary material

Table S1. Structural alignment of the *Mt*KARI open and close states to the structurally characterised non-mutant KARIs. A green to red colour code represents lowest to highest rmsd. *Mt, Methanothermococcus thermolithotrophicus; Ala, Alicyclobacillus acidocaldarius; Slae, Slackia exigua; Azv, Azotobacter vinelandii; Pa, Pseudomonas aeruginosa; Ua, Uncultured archeon, Staa, Staphylococcus aureus; Ia, Ignisphaera aggregans; Strp, Streptococcus pneumoniae; Myt, Mycobacterium tuberculosis; Sula, Sulfolobus acidocaldarius; Cg, Corynebacterium glutamicum; Sacs, Saccharolobus solfataricus (previously Sulfolobus solfataricus); Ec, Escherichia coli.*

	Class	Seq. Identity	Ligand (3-letter code)	*Mt*KARI close state rmsd (aligned Cα)	*Mt*KARI open state rmsd (aligned Cα)
Close *Mt*KARI (7Q03*)*	I	100	MG, NDP	0 (**328**)	1.159 (**283**)
Open *Mt*KARI (7Q07)	I	100	NDP, TLA	1.160 (**283**)	0 (**327**)
*Ala*KARI (4TSK)	I	56.53 %	MG, TLA, NDP	0.599 (**309**)	1.590 (**299**)
*Slae*KARI (4KQW)	I	53.56 %	MG, TLA, NAP	0.676 (**295**)	1.706 (**298**)
*Azv*KARI (4XIY)	I	52.94 %	MG, FE	1.337 (**262**)	0.853 (**288**)
*Pa*KARI (1NP3)	I	51.08 %	-	1.258 (**256**)	0.894 (**297**)
*Ua*KARI (4XDY)	I	51.52 %	MG, NAI, HIO	0.701 (**303**)	1.651 (**305**)
*Staa*KARI (6VO2)	I	52.58 %	MG, NDP, R67	0.74 (**298**)	1.628 (**300**)
*Staa*KARI (5W3K)	I	52.58 %	MG, NDP, 9TY	0.687 (**294**)	1.615 (**299**)
*Staa*KARI (6AQJ)	I	52.58 %	MG, NDP, HIO, 40E	0.789 (**297**)	1.739 (**305**)
*Ia*KARI (4XDZ)	I	51.52 %	MG, NDP, 40E	0.759 (**260**)	1.779 (**288**)
*Strp*KARI (6L2I)	I	53.14 %	MG, NAP	1.360 (**272**)	0.731 (**290**)
*Myt*KARI (4YPO)	I	51.09 %	MG	1.492 (**275**)	0.913 (**312**)
*Sula*KARI (5YEQ)	I	50.46 %	MG	0.931 (**252**)	0.868 (**288**)
*Cg*KARI (6JX2)	I	48.92 %	MG, NAP	1.539 (**296**)	0.634 (**284**)
*Sacs*KARI (6JCV)	I	36.70 %	MG	2.226 (**244**)	1.326 (**236**)
*Sacs*KARI (6JCZ)	I	36.70 %	MG, NDP, 9TY	2.400 (**254**)	1.491 (**245**)
*Ec*KARI (1YRL)	II	36.30 %	-	1.068 (**192**)	1.083 (**198**)

Table S2. Structural alignment of the structure of KARIs from *C. glutamicum* and *S. pneumoniae* to the structurally characterised non-mutant KARIs. A green to red colour code represents lowest to highest rmsd and the name nomenclature is the same as in Table S1.

	Ligand (3-letter code)	*Cg*KARI (6JX2) rmsd (aligned Cα)	*Strp*KARI (6L2I) rmsd (aligned Cα)
Close *Mt*KARI (7Q03)	MG, NDP	1.539 (296)	1.360 (**272**)
Open *Mt*KARI (7Q07)	NDP, TLA	0.634 (**284**)	0.731 (**290**)
*Ala*KARI (4TSK)	MG, TLA, NDP	1.662 (**296**)	1.662 (**287**)
*Slae*KARI (4KQW)	MG, TLA, NAP	1.915 (**313**)	1.799 (**293**)
*Azv*KARI (4XIY)	MG, FE	0.806 (**271**)	0.934 (**287**)
*Pa*KARI (1NP3)	-	0.965 (**294**)	0.860 (**271**)
*Ua*KARI (4XDY)	MG, NAI, HIO	1.724 (**308**)	1.705 (293)
*Staa*KARI (6VO2)	MG, NDP, R67	1.778 (305)	1.868 (**301**)
*Staa*KARI (5W3K)	MG, NDP, 9TY	1.749 (302)	1.873 (302)
*Staa*KARI (6AQJ)	MG, NDP, HIO, 40E	1.767 (302)	1.805 (**292**)
*Ia*KARI (4XDZ)	MG, NDP, 40E	1.763 (**287**)	1.547 (**257**)
*Strp*KARI (6L2I)	MG, NAP	0.808 (**282**)	0 (330)
*Myt*KARI (4YPO)	MG	0.964 (318)	0.789 (271)
*Sula*KARI (5YEQ)	MG	0.832 (**280**)	1.112 (**293**)
*Cg*KARI (6JX2)	MG, NAP	0 (325)	0.808 (**282**)
*Sacs*KARI (6JCV)	MG	1.482 (**237**)	1.363 (**236**)
*Sacs*KARI (6JCZ)	MG, NDP, 9TY	1.575 (**241**)	1.413 (**241**)
*Ec*KARI (1YRL)	-	1.930 (**189**)	1.148 (227)

Table S3. Oligomeric state of KARI. The name nomenclature is the same as in Table S1.

	Class	Sequence Identity	Proposed Oligomeric state	Experimental validation	Reference
*Mt*KARI (7Q03*)*	I	100	Dodecamer	Native electrophoresis, Size exclusion chromatography	This study
*Ala*KARI (4TSK)	I	56.53 %	Dimer	-	Cahn et al., 2015 [1]
*Slae*KARI (4KQW)	I	53.56 %	Dimer	-	Brinkmann-Chen et al., 2013 [2]
*Azv*KARI (4XIY)	I	52.94 %	Dimer	-	Cahn et al., 2015 [1]
*Pa*KARI (1NP3)	I	51.08 %	Dodecamer	Size exclusion chromatography, dynamic light-scattering	Ahn et al., 2003 [3]
*Ua*KARI (4XDY)	I	51.52 %	Dimer	-	Cahn et al., 2015 [1]
*Staa*KARI (6VO2)	I	52.58 %	Dimer	Size exclusion chromatography	Patel et al., 2017 [4]
*Ia*KARI (4XDZ)	I	51.52 %	Dimer	-	Cahn et al., 2015 [1]
*Strp*KARI (6L2I)	I	53.14 %	Dimer	Small-angle X-ray scattering	Kim et al., 2019 [5]
*Myt*KARI (4YPO)	I	51.09 %	Dimer	Multi-angle light scattering	Lv et al., 2016 [6]
*Sula*KARI (5YEQ)	I	50.46 %	Dimer	Size exclusion chromatography	Chen et al., 2018 [7]
*Cg*KARI (6JX2)	I	48.92 %	Dimer	Size exclusion chromatography	Lee et al., 2019 [8]
Helicobacter pylori KARI (WP_001207734.1)	I	42.24 %	Dodecamer	Dynamic light-scattering	Ahn et al., 2003 [3]
*Sacs*KARI (6JCV)	I	36.70 %	Dodecamer	Size exclusion chromatography	Chen et al., 2019 [9]
*Ec*KARI (1YRL)	II	36.30 %	Tetramer	-	Tyagi et al., 2005 [10]

Table S4. Oligomeric state of KARIs depending on optimal growing conditions of the organisms

	KARI Oligomeric state	Optimal temperature for growth	Additional salt in medium	Optimal pH for growth	Reference
M. thermolithotrophicus	Dodecamer	60 °C	0.5 M	7	Huber et al. 1982 [11]
P. aeruginosa	Dodecamer	37 °C	No	7.2	LaBauve and Wargo, 2012 [12]
S. aureus	Dimer	37 °C	No	7.2	Missiakas and Schneewind 2013 [13]
S. pneumoniae	Dimer	37 °C	No	7.2	Suárez and Texeira, 2019 [14]
M. tuberculosis	Dimer	37 °C	No	7.2	Martin et al, 1975 [15]
S. acidocaldarius	Dimer	75 °C	No	3	Quehenberger et al. 2019 [16]
C. glutamicum	Dimer	30 °C	No	8.5	Jakob et al 2007 [17]
H. pylori	Dodecamer	37 °C	No	7	Blanchard and Nedrud., 2006 [18]
S. sulfataricus	Dodecamer	75-80 °C	No	2-3	Park and Lee, 1999 [19]

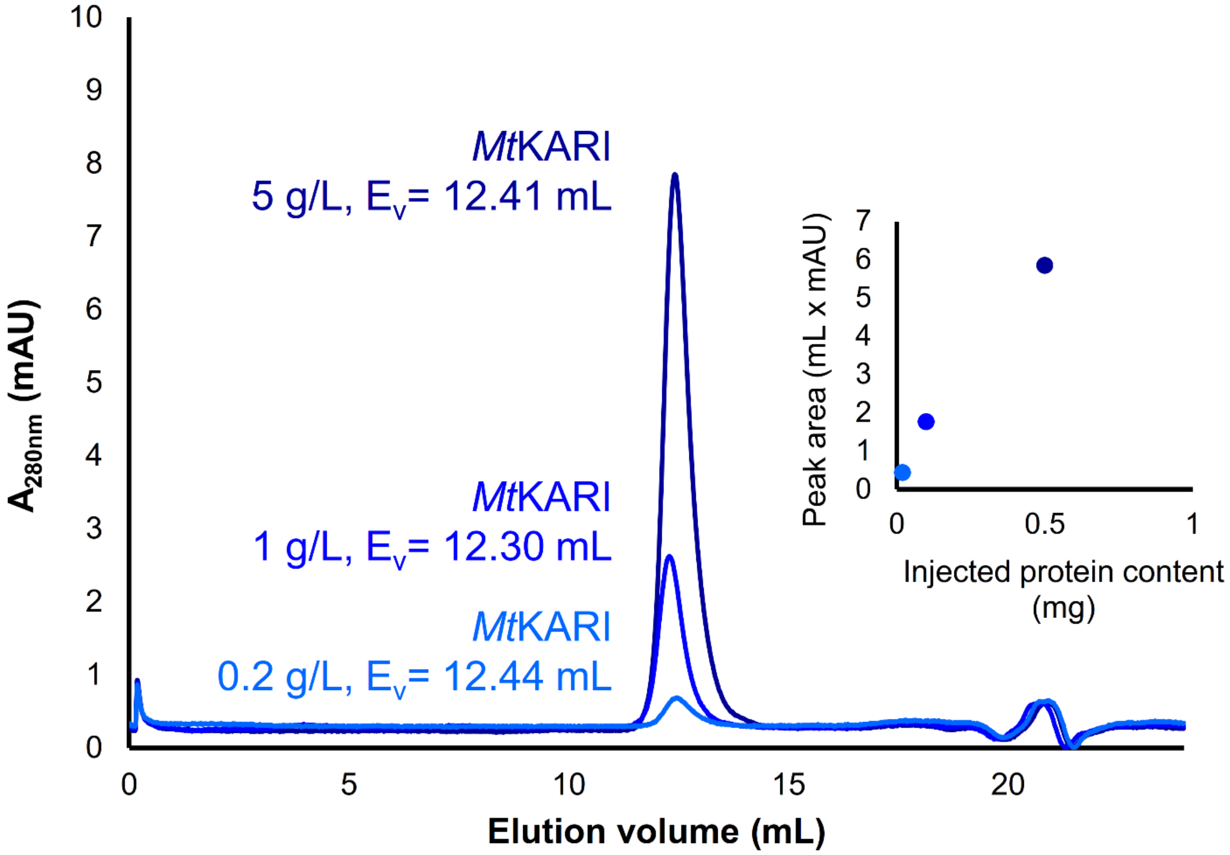

Figure S1. Size exclusion chromatography of *Mt*KARI at different protein concentrations. Elution chromatograms (coloured in blue shades) of *Mt*KARI injected at different protein concentrations. For this experiment, 100 µL sample volume was used. The elution volumes (E_V) are indicated and gathered in Figure 1D. An insert presents the peak areas depending on the loaded protein amount.

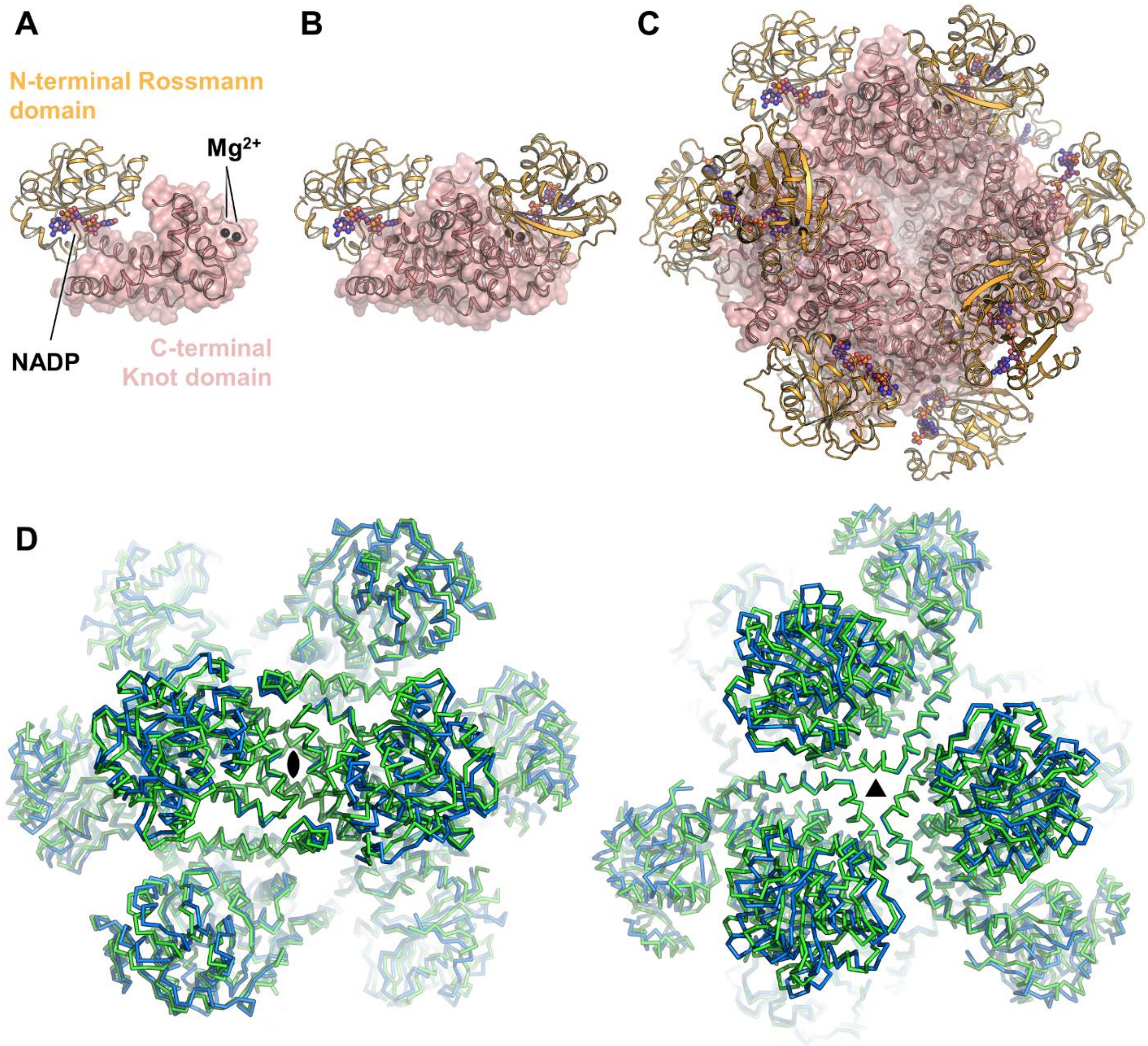

Figure S2. Domain organisation and assembly. Cartoon representation of the monomeric (**A**), dimeric (**B**) and dodecameric (**C**) organisation of *Mt*KARI. The Rossmann domain is coloured in orange and the knot domain, highlighted by a transparent surface, is coloured in pink. (**D**) Overall superposition of the close (green) and open (blue) states of dodecameric *Mt*KARI. Left panel, view along the 2-fold symmetry axis. Right panel, view along the 3-fold symmetry axis.

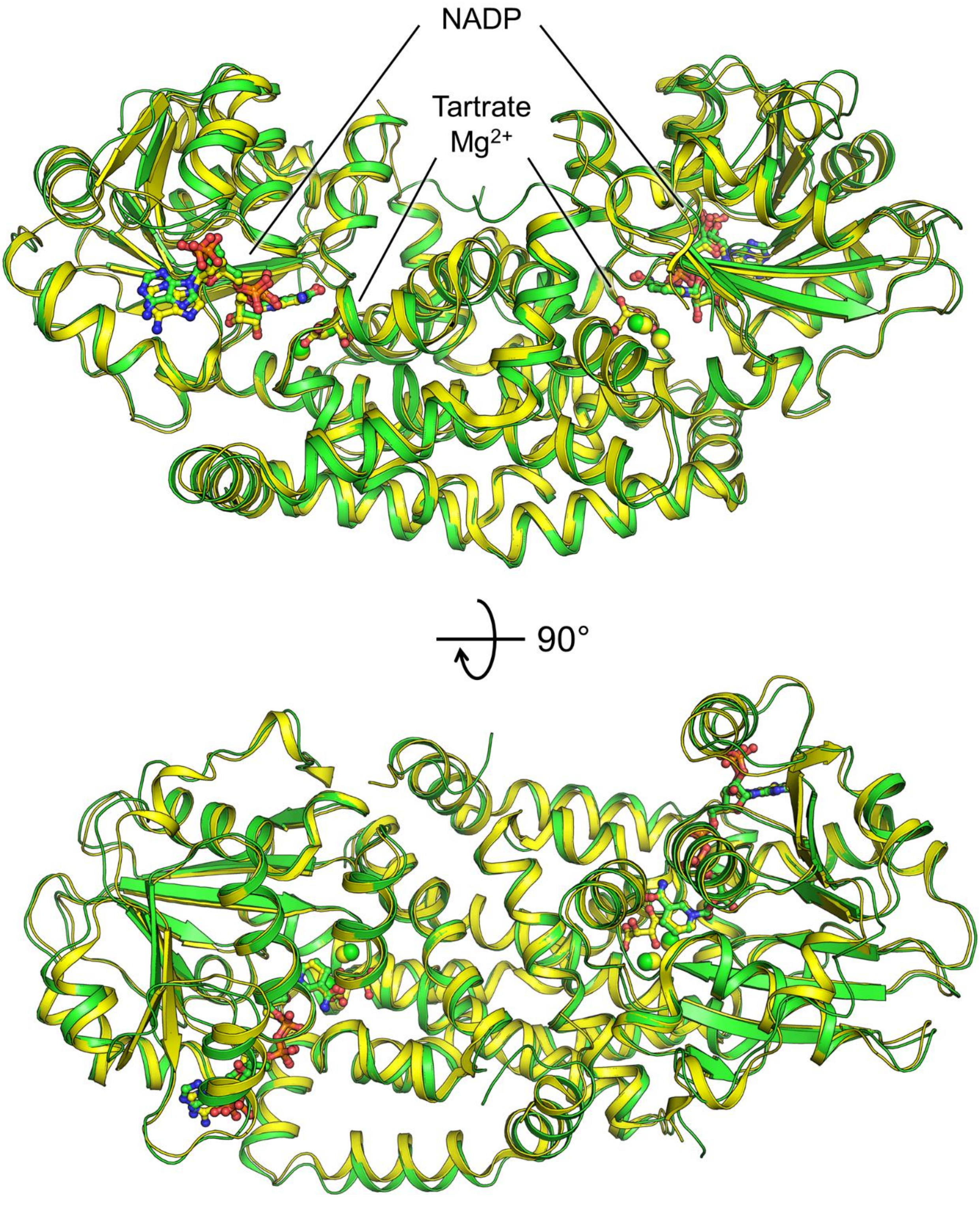

Figure S3. Superposition of the structures of close *Mt*KARI and *Slae*KARI (4KQW). Superposition of close *Mt*KARI (green cartoon) and close *Slae*KARI (yellow cartoon). Ligands are represented by balls and sticks with oxygen, nitrogen, phosphorus and Mg^{2+} coloured in red, blue, orange and light green, respectively. The carbon atoms have the same colours as their models.

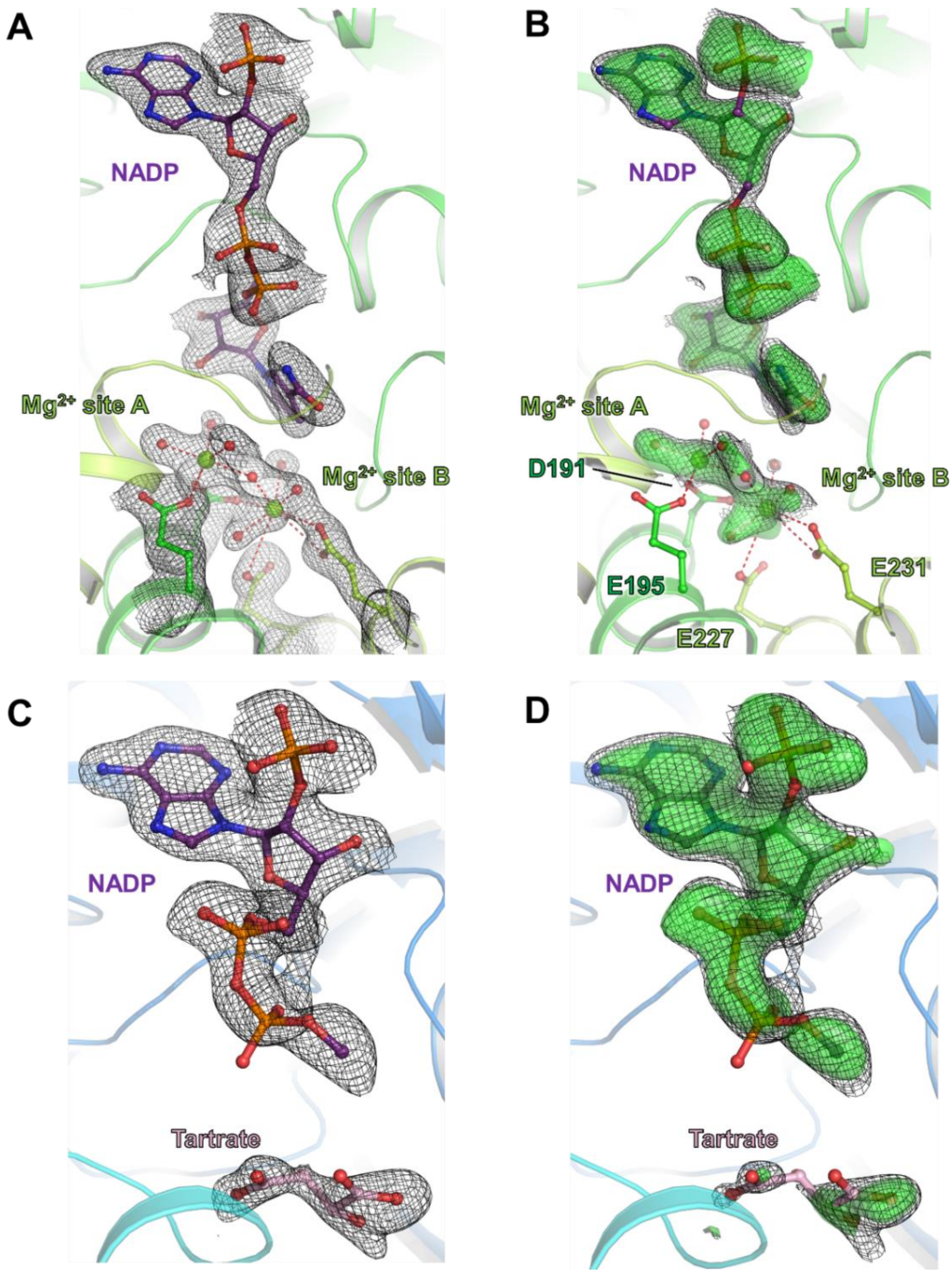

Figure S4. Electron density and omit map of Mg^{2+}, NADP, and tartrate in *Mt*KARI structures. (**A**) The $2F_o$-F_c map, shown as black mesh is contoured at 1-σ for the NADP and Mg^{2+} binding site in the close state. (**B**) Omit map for the NADP, Mg^{2+} and its water network in the close state. The $2F_o$-F_c omit map (black mesh) is contoured at 1-σ and F_o-F_c omit map (transparent green surface) is contoured at 3-σ. (**C**) The $2F_o$-F_c map, shown as black mesh is contoured at 1-σ for the NADP and tartrate in the open state. (**D**) Omit map for the NADP and tartrate in the open state. The $2F_o$-F_c omit map (black mesh) is contoured at 1-σ and F_o-F_c omit map (transparent green surface) is contoured at 3-σ. For all panels, ligands are shown in balls and sticks with oxygen, nitrogen, phosphorus and Mg^{2+} coloured in red, blue, orange and light green, respectively. Carbons are coloured in purple and pink for the NADP and tartrate, respectively.

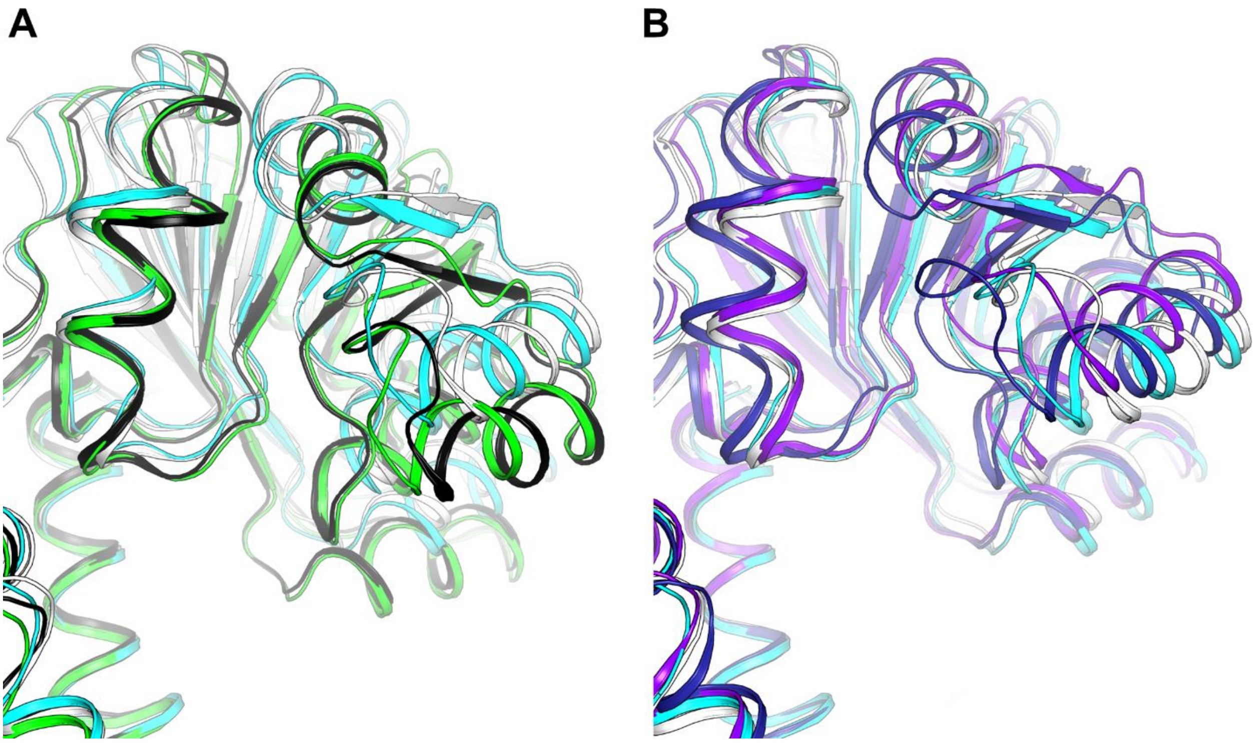

Figure S5. Structural alignment of open and close state KARI structures. (A-B) Structures are aligned on the knot domain and represented as cartoon. (**A**) Structural alignment of the close *Mt*KARI (green), the open *Mt*KARI (cyan), the close *Slae*KARI (PDB ID: 4KQW, black) and the open *Pa*KARI (PDB ID: 1NP3, white). (**B**) Structural alignment of *Strp*KARI (PDB ID: 6L2I, purple) and *Cg*KARI (PDB ID: 6JX2, dark blue) to the open *Pa*KARI and *Mt*KARI, coloured as in A.

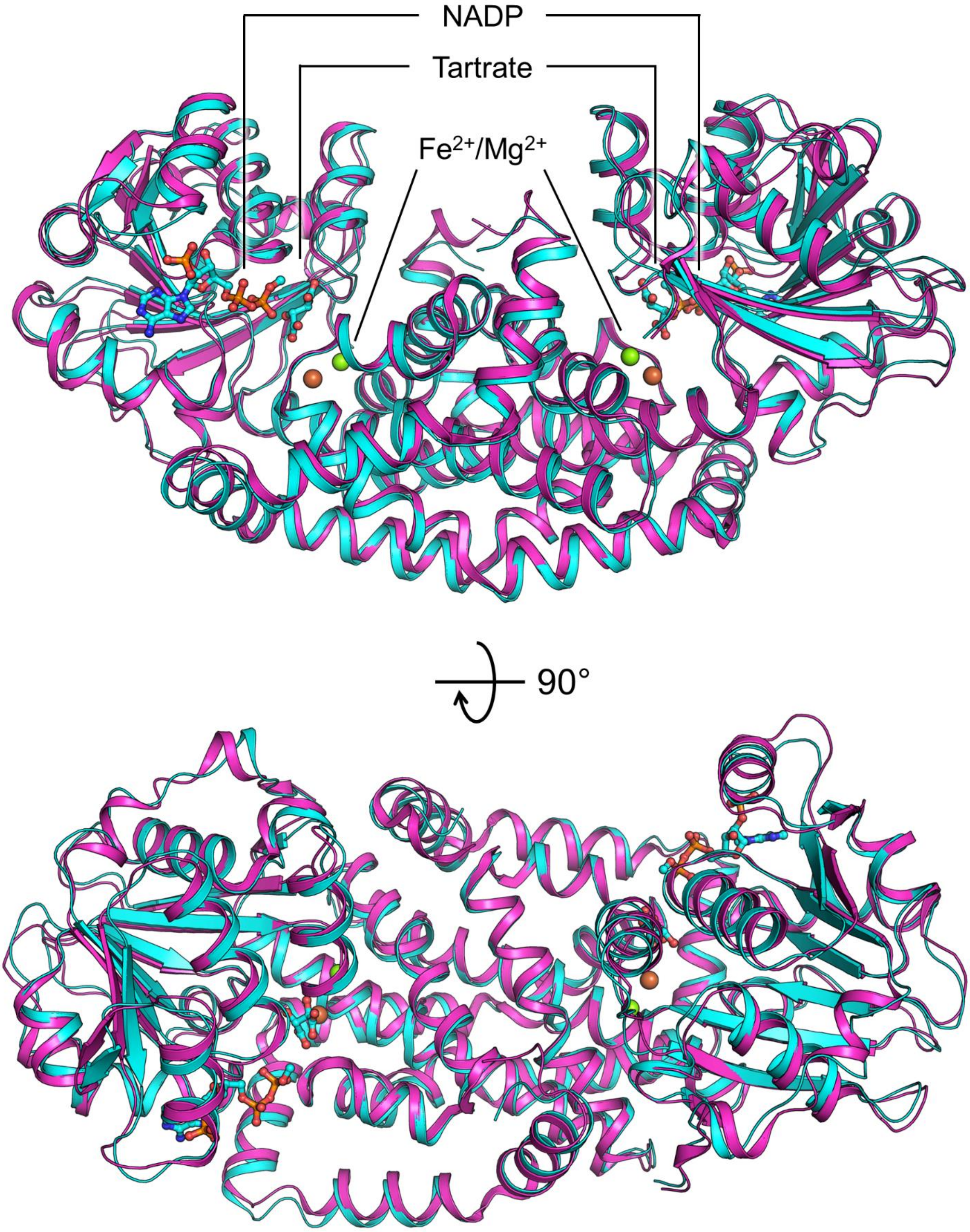

Figure S6. Superposition of the structures of open *Mt*KARI and *Azv*KARI (4XIY). Superposition of open *Mt*KARI (cyan cartoon) and *Azv*KARI (magenta cartoon). Ligands are represented by balls and sticks with oxygen, nitrogen and phosphorus coloured in red, blue, orange and light green, respectively. The carbon atoms are coloured as the model. Fe and Mg atoms from *Azv*KARI are shown as balls and coloured in green and orange, respectively.

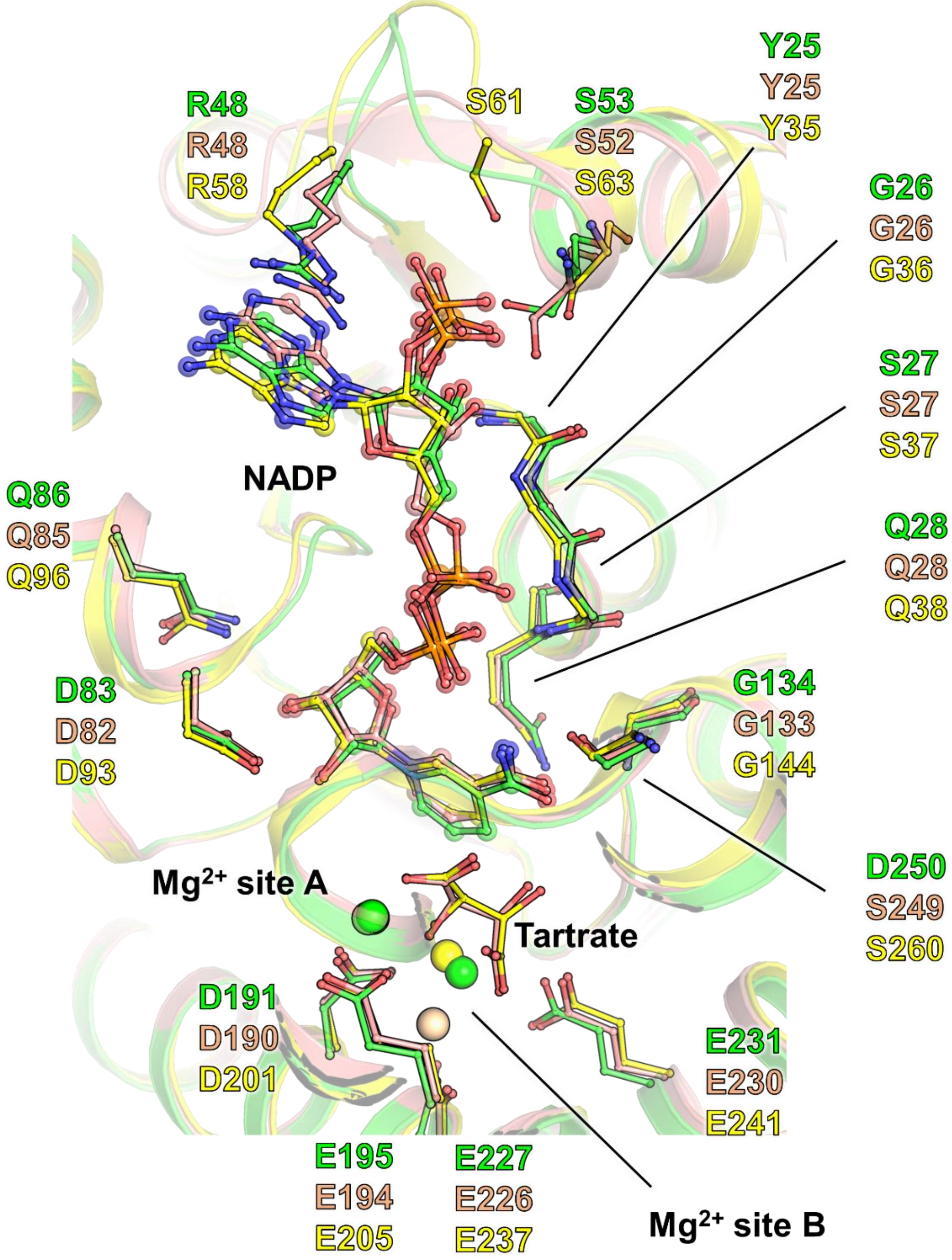

Figure S7. Colocalisation of NADP and Mg^{2+} in close state structures of *Mt*KARI, *Ala*KARI (4TSK) and *Slae*KARI (4KQW). The structures are represented in cartoon and coloured in green (*Mt*KARI), wheat (*Ala*KARI) and yellow (*Slae*KARI). Ligands and interacting residues are represented by balls and sticks and coloured in red, blue, and orange for oxygen, nitrogen and phosphorus, respectively. Carbons and Mg^{2+} ions (as spheres) are coloured according to the model. NADP is highlighted by transparent spheres. Tartrate is not present in the close state of *Mt*KARI.

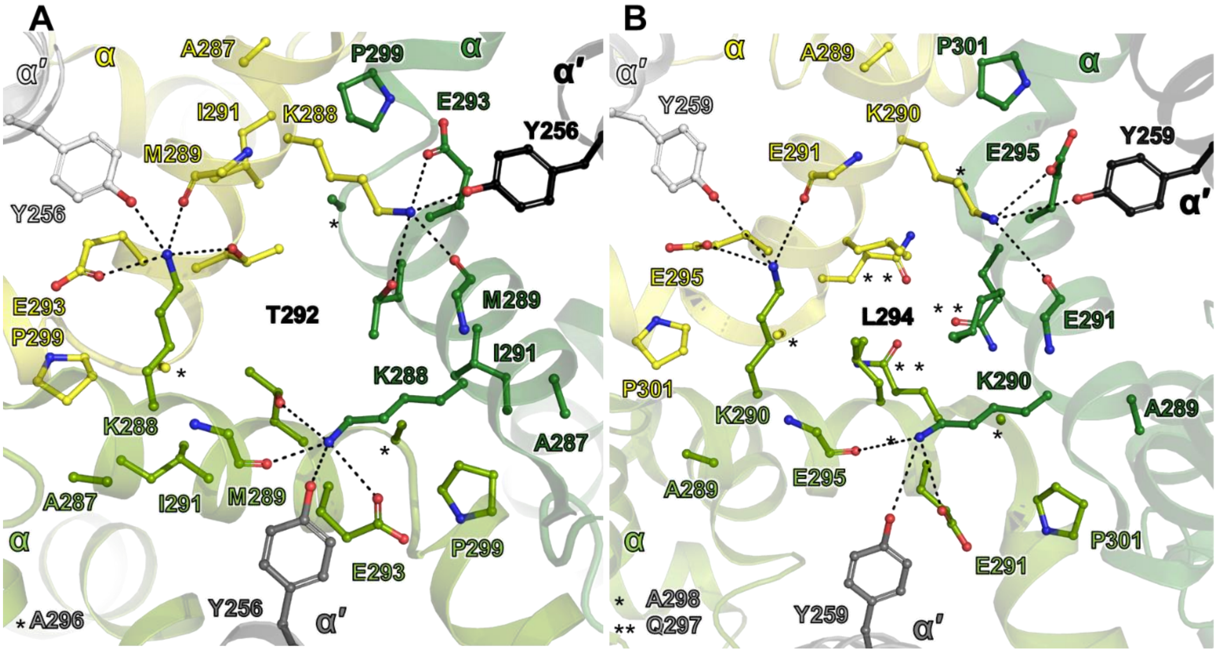

Figure S8. Trimeric interface in *Pa*KARI (1NP3) and *Sacs*KARI (6KPJ). Close up of the trimeric interface area. The structures are represented in cartoon and coloured per monomer. Residues stabilising the interface are represented by sticks and coloured with oxygen, nitrogen, and sulphur in red, blue and yellow, respectively. Hydrogen bonds are represented by dashed lines. Hydrophobic residues (Ala296, Ala287, Ile291, *Pa*KARI numbering) and Pro299 (*Pa*KARI) compose the “hydrophobic brace” proposed by Lv et al (2016) [6].

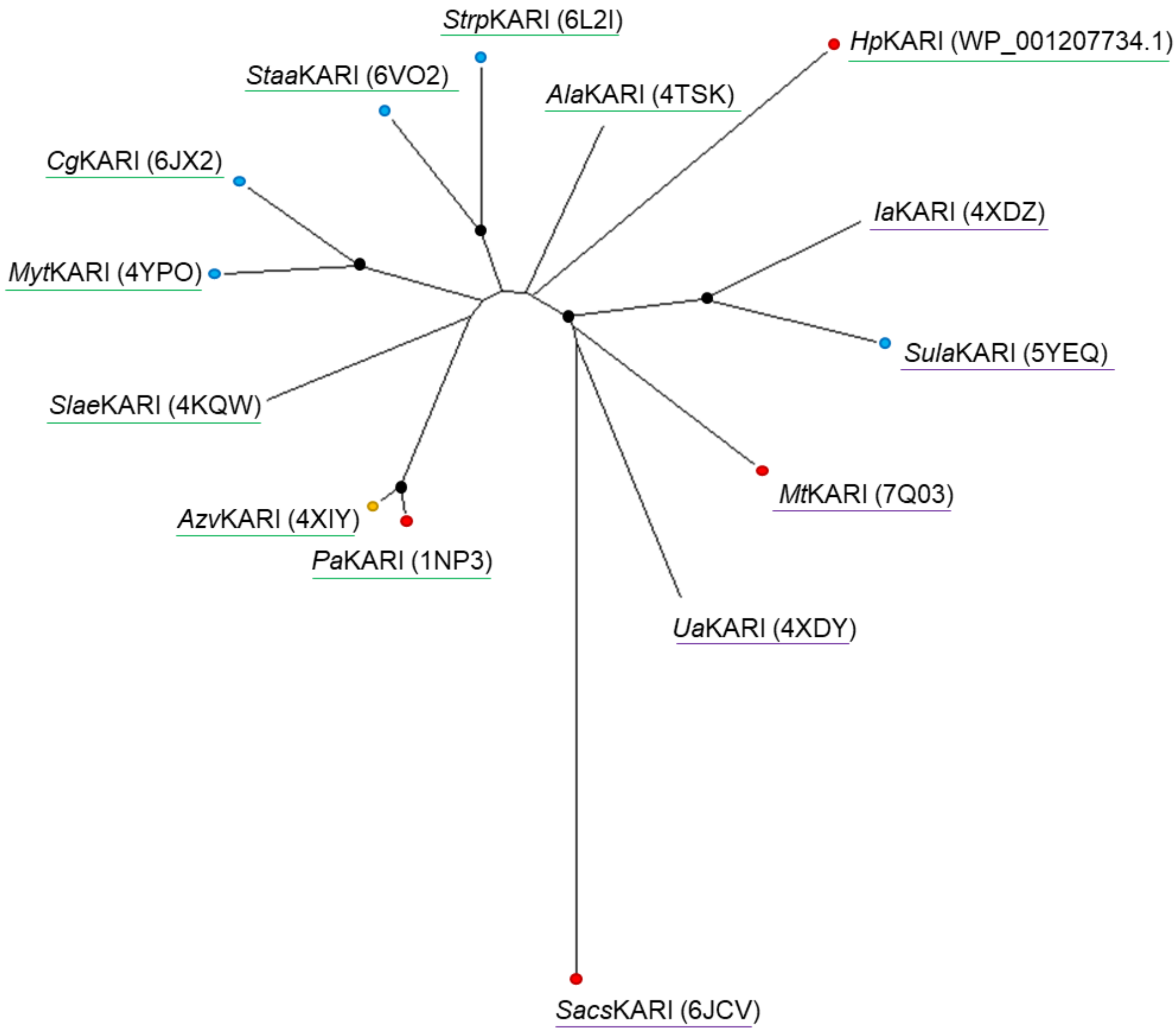

Figure S9. Evolutionary relationships of the characterised KARIs used in this work. The evolutionary history was inferred using the Minimum Evolution method and the tree was constructed by the MEGA program [20]. Nodes with a score higher than 95 % (2000 replicates) are shown as black circles. Experimentally proven dimeric and dodecameric KARIs are indicated as blue and red circles, respectively. An orange circle indicates the putative dodecameric *Azv*KARI [1,6]. Enzymes originally from archaea and bacteria are underlined in purple and green, respectively.

References 1-20

1. Cahn, J.K.B.; Brinkmann-Chen, S.; Spatzal, T.; Wiig, J.A.; Buller, Andrew R.; Einsle, O.; Hu, Y.; Ribbe, M.W.; Arnold, F.H. Cofactor specificity motifs and the induced fit mechanism in class I ketol-acid reductoisomerases. *Biochemical Journal* **2015**, *468*, 475-484.
2. Brinkmann-Chen, S.; Flock, T.; Cahn, J.K.B.; Snow, C.D.; Brustad, E.M.; McIntosh, J.A.; Meinhold, P.; Zhang, L.; Arnold, F.H. General approach to reversing ketol-acid reductoisomerase cofactor dependence from NADPH to NADH. *Proc Natl Acad Sci U S A* **2013**, *110*, 10946-10951.
3. Ahn, H.J.; Eom, S.J.; Yoon, H.J.; Lee, B.I.; Cho, H.; Suh, S.W. Crystal structure of class I acetohydroxy acid isomeroreductase from *Pseudomonas aeruginosa*. *J Mol Biol* **2003**, *328*.
4. Patel, K.M.; Teran, D.; Zheng, S.; Kandale, A.; Garcia, M.; Lv, Y.; Schembri, M.A.; McGeary, R.P.; Schenk, G.; Guddat, L.W. Crystal Structures of *Staphylococcus aureus* Ketol-Acid Reductoisomerase in Complex with Two Transition State Analogues that Have Biocidal Activity. *Chemistry* **2017**, *23*, 18289-18295.
5. Kim, G.; Shin, D.; Lee, S.; Yun, J.; Lee, S. Crystal Structure of IlvC, a Ketol-Acid Reductoisomerase, from *Streptococcus Pneumoniae*. *Crystals* **2019**, *9*, 551.
6. Lv, Y.; Kandale, A.; Wun, S.J.; McGeary, R.P.; Williams, S.J.; Kobe, B.; Sieber, V.; Schembri, M.A.; Schenk, G.; Guddat, L.W. Crystal structure of *Mycobacterium tuberculosis* ketol-acid reductoisomerase at 1.0 Å resolution - a potential target for anti-tuberculosis drug discovery. *FEBS J* **2016**, *283*, 1184-1196.
7. Chen, C.Y.; Ko, T.P.; Lin, K.F.; Lin, B.L.; Huang, C.H.; Chiang, C.H.; Horng, J.C. NADH/NADPH bi-cofactor-utilizing and thermoactive ketol-acid reductoisomerase from *Sulfolobus acidocaldarius*. *Scientific Reports* **2018**, *8*, 7176.
8. Lee, D.; Hong, J.; Kim, K.J. Crystal Structure and Biochemical Characterization of Ketol-Acid Reductoisomerase from *Corynebacterium glutamicum*. *J Agric Food Chem* **2019**, *67*, 8527-8535.
9. Chen, C.Y.; Chang, Y.C.; Lin, B.L.; Lin, K.F.; Huang, C.H.; Hsieh, D.L.; Ko, T.P.; Tsai, M.D. Use of Cryo-EM To Uncover Structural Bases of pH Effect and Cofactor Bispecificity of Ketol-Acid Reductoisomerase. *J Am Chem Soc* **2019**, *141*, 6136-6140.
10. Tyagi, R.; Duquerroy, S.; Navaza, J.; Guddat, L.W.; Duggleby, R.G. The crystal structure of a bacterial class II ketol-acid reductoisomerase: domain conservation and evolution. *Protein Sci* **2005**, *14*, 3089-3100.
11. Huber, H.; Thomm, M.; König, H.; Thies, G.; Stetter, K.O. *Methanococcus thermolithotrophicus*, a novel thermophilic lithotrophic methanogen. *Archives of Microbiology* **1982**, *132*, 47-50.
12. LaBauve, A.E.; Wargo, M.J. Growth and Laboratory Maintenance of *Pseudomonas aeruginosa*. *Current Protocols in Microbiology* **2012**, *25*, 6E.1.1-6E.1.8.
13. Missiakas, D.M.; Schneewind, O. Growth and laboratory maintenance of *Staphylococcus aureus*. *Curr Protoc Microbiol* **2013**, *Chapter 9*, Unit 9C.1.
14. Suárez, N.; Texeira, E. Optimal Conditions for *Streptococcus pneumoniae* Culture: In Solid and Liquid Media. *Methods Mol Biol* **2019**, *1968*, 3-10.
15. Martin, R.S.; Sumarah, R.K.; Robart, E.M. Comparison of four culture media for the isolation of *Mycobacterium tuberculosis*: a 2-year study. *J Clin Microbiol* **1975**, *2*, 438-440.
16. Quehenberger, J.; Albersmeier, A.; Glatzel, H.; Hackl, M.; Kalinowski, J.; Spadiut, O. A defined cultivation medium for *Sulfolobus acidocaldarius*. *J Biotechnol* **2019**, *301*, 56-67.
17. Jakob, K.; Satorhelyi, P.; Lange, C.; Wendisch, V.F.; Silakowski, B.; Scherer, S.; Neuhaus, K. Gene expression analysis of *Corynebacterium glutamicum* subjected to long-term lactic acid adaptation. *J Bacteriol* **2007**, *189*, 5582-5590.
18. Blanchard, T.G.; Nedrud, J.G. Laboratory maintenance of *Helicobacter* species. *Current protocols in microbiology* **2006**, *Chapter 8*, Unit8B.1-Unit8B.1.
19. Park, C.B.; Lee, S.B. Inhibitory effect of mineral ion accumulation on high density growth of the hyperthermophilic archaeon *Sulfolobus solfataricus*. *Journal of Bioscience and Bioengineering* **1999**, *87*, 315-319.
20. Kumar, S.; Stecher, G.; Li, M.; Knyaz, C.; Tamura, K. *MEGA X*: Molecular Evolutionary Genetics Analysis across Computing Platforms. *Molecular biology and evolution* **2018**, *35*, 1547-1549.
